# Unraveling the Complexities of Flowering in Ornamental Plants: The Interplay of Genetics, Hormonal Networks, and Microbiome

**DOI:** 10.3390/plants14071131

**Published:** 2025-04-06

**Authors:** Muhammad Aizaz, Lubna, Syed Salman Hashmi, Muhammad Aaqil Khan, Rahmatullah Jan, Saqib Bilal, Kyung-Min Kim, Ahmed Al-Harrasi, Sajjad Asaf

**Affiliations:** 1 Natural and Medical Science Research Center, University of Nizwa, Nizwa 616, Oman; 2 Department of Chemical and Life Science, Qurtaba University of Science and Technology, Peshawar 25000, Pakistan; aqil_bacha@yahoo.com; 3 Coastal Agriculture Research Institute, Kyungpook National University, Daegu 41566, Republic of Korea; 4 Department of Applied Biosciences, Kyungpook National University, Daegu 41566, Republic of Korea

**Keywords:** plant microbiome interaction, flower induction, *FT*, *SOC1*, *LFY* genes, plant hormones

## Abstract

In ornamental plants, one of the most complex life processes, i.e., flowering, is regulated by interaction between the microbiota, hormones, and genes. Flowering plays an integral role in overall development and is quintessential for reproduction. Considering its importance, this review explores the complex mechanisms that determine the induction of flowering, highlighting the relationship between hormonal and genetic networks as well as the growing significance of the microbiome. Important genes involved in genetic control include *FT*, *SOC1*, and *LFY*. These genes react to environmental stimuli like photoperiod and vernalization. Auxins, cytokinin, and gibberellins are only a few hormone pathways important for floral growth and timing. The importance of plant–microbe interactions has been emphasized by current research, which shows that the microbiome affects flowering through processes like hormone production and availability of food. A comprehensive understanding of flowering induction is possible by integrating results from microbiota, hormones, and genetics studies, which may improve the breeding and culture of ornamental plants. For researchers to understand the complexity of flowering in ornamental plants and develop unique breeding strategies and improved floral qualities, it is critical to use interdisciplinary approaches, as this comprehensive investigation demonstrates.

## 1. Introduction

### 1.1. Brief Overview of the Importance of Flowering in Ornamental Plants

Ornamental plants are the most diverse products in horticulture and belong to a rapidly developing industry [1]. They consist of a vast and diverse range of whole plants or plant parts primarily cultivated for decorative purposes [2]. Nowadays, the global ornamental plant industry is characterized by significant growth in both production and consumption, contributing to globalization and international trade [3]. Major markets have become increasingly interconnected, leading to greater seasonality and variability in the supply and demand for plants and flowers, which has contributed to enhanced price volatility [3]. Europe is one of the major markets for ornamentals and is expected to have one of the best growth rates in both production and consumption over the next ten years [4]. Demand for ornamental plants in Europe has shown a significant increase in purchases and price premiums across both the institutional and private sectors [5].

Ornamental plants provide apparent delight through their colorful and differently shaped flowers, fruits, and leaves, making ’beauty’ a major ornamentation trait [6]. Plants play a significant role in various sectors because of their importance and versatility. Multifaceted uses of ornamental plants include floristry, landscaping, gardening, potted plants, and cut flowers [7]. Flowers alone have played a significant role in human society. Their multifaceted applications in medicine, culinary arts, psychological well-being, and mental health have been well-noted. Studies demonstrate various functions of flowers, including their potential for food production and agriculture, pharmaceutical applications, health benefits, and environmental remediation [8]. Flowers have medicinal properties that help reduce anxiety, headaches, and memory loss. They also help to maintain physical health by treating digestive disorders and stomach ulcers [9]. Flowers are employed in the culinary arts to enhance the visual appeal of dishes and provide different dining experiences [10]. Flowers benefit mood and emotional wellness, as evidenced by a study demonstrating that examining photos of flowers makes one feel cheerful. This reflects the significance of ornamental flowers for enhancing general happiness and well-being [11].

**Figure 1:**
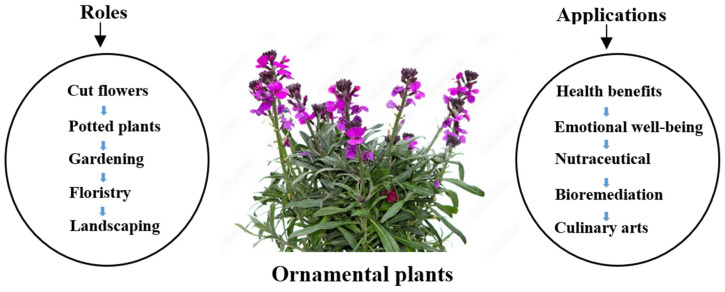
Different roles and applications of ornamental plants.

Additionally, edible flowers are considered a diverse and vital source of essential vitamins, antioxidants, and medicinal pigments. They also have the potential to be used as nutraceuticals for supplemental health products, as shown in Figure 1 [12]. The demand for edible flowers has increased for several reasons, including growing knowledge of their beneficial effects and nutritional value [13]. Edible flowers contain numerous bioactive compounds, each with particular health benefits, such as vitamins, carotenoids, flavonoids, and phenolics [13]. The increased demand for functional food items and the shift in eating habits towards a healthier diet have also contributed to the increasing popularity of edible flowers [14]. Furthermore, the development of special horticulture products, such as edible flowers, has made it easier to expand diets and meet the demand of consumers for useful and healthy foods, which has spurred the development of this particular market sector [15].

### 1.2. Introduction to the Genetic and Hormonal Networks Regulating Flowering

Plants flower due to complex hormonal and genetic processes that combine internal and external stimuli to control the transition from vegetative to reproductive stages. Several studies have explored these systems, such as the *Arabidopsis thaliana* Flowering Transition Gene Regulatory Network (FT-GRN), which combines intrinsic and extrinsic cues to trigger shoot apical meristem (SAM) phase modifications [16]. Research on *Arabidopsis thaliana* has shown the crucial functions performed by important genes, such as blooming Flowering Locus C (*FLC*) and Flowering Locus T (*FT*), in the regulatory system that controls the blooming process [17]. Moreover, microRNAs like miR156 and miR172 are part of regulatory circuits that control how environmental factors like CO_2_ levels affect the flowering time [18]. Gene-centric GWAS approaches have also highlighted the significance of gene interactions in controlling flowering time, as demonstrated by gene–phenotype association studies that have identified many genes associated with flowering time [19]. These genetic networks control blooming time and show intricate interactions between multiple genes and environmental stimuli, highlighting the flexibility and complexity of the mechanisms controlling flowering time in plants.

On the other hand, hormones like gibberellins (GA), auxin, cytokinins, abscisic acid (ABA), and ethylene also play important roles in controlling blooming [20]. Studying species like *Rosa rugosa* showed how phytohormones, starch, and sugars function during the changing process from vegetative to floral state, demonstrating the intricate gene-to-floral interaction process [21]. Plant hormones are typically essential to regulating orchid flowering [22]. Auxin is a morphogen [23] that serves as a signal for the specification of plant tissues based on its concentration gradient [24]. In *Dendrobium* and *Phalaenopsis* orchids, applying 6-benzylaminopurine (BA), a synthetic cytokinin, promotes flowering. This effect is mitigated by auxin. Exogenous BA is introduced into *Phalaenopsis* and *Doritaenopsis* plants to induce an early flowering time [25]. The limitation of *Petunia hybrida* flower development triggered by high temperatures can be alleviated by injecting GAs [26]. When GA (GA3) and BA are applied together, flowering has a noticeable effect [27]. GA also regulates essential processes like blooming timing and stem elongation [28]. Bud break and blooming period are both regulated by ABA [29]. It is hypothesized that strigolactones interact with GA, ethylene, auxins, and cytokinins to influence blooming [30]. ABA controls bud dormancy via the photoperiodic pathway [31]. It also inhibits glucanase activity while increasing the expression of Callose Synthase 1 (*CALS1*). This results in using dormancy sphincters that close intercellular channels (plasmodesmata) by inhibiting the flow of growth-promoting stimuli that cause dormancy [32]. The transcriptome of *Arundina graminifolia* included several homologs of *CALS*. The expression was significantly upregulated in the first stage of flower bud emergence. Furthermore, in the initial phases of bud development, the ABA-responsive ABFs showed significant expression levels, demonstrating that ABA influences the temporal regulation of bud growth [33]. Studies on the level of ABA in different *Phalaenopsis* tissues have shown that the dormant axillary buds contain significant amounts of free ABA [34]. Moreover, ABA inhibits the development of floral spikes when applied externally to the stem of *Phalaenopsis*, suggesting that ABA has an inhibitory effect on the transition of orchid flowers [34]. However, additional thorough research studies will be required to understand the role of ABA and other key hormones in regulating orchid flowering.

### 1.3. Microbial Diversity and Its Impact on Floral Biology and Pollination

Several scientific studies contribute to the potentially rising appeal of floral microbiomes [35]. Searching for rare yeast strains has demonstrated that flowers and the insects pollinating them are important biodiversity sources [36]. Nectar microcosms have been employed in community ecology to investigate factors influencing community assembly [37]. Within the field of conservation biology, there has been a notable decline in pollinators, alongside a growing acknowledgment of the significance of pathogens. Consequently, this has resulted in categorizing flowers as key sites for transmitting crucial pollinator pathogens [38]. Biocontrol compounds produced from floral surfaces have demonstrated significance in plant pathology for addressing floral diseases and enhancing orchard sustainability in pathogen management [39]. All these different studies combined are the demonstration of significance of floral microbiota as indispensable model for research into evolutionary and ecological processes.

Fungi and bacteria are already present while a bloom is just starting to develop. Both the nectar inside closed flowers and the flower buds individually may contain recognizable bacterial and fungal species before anthesis, the stage at which the flower opens [40]. Microbial communities that can be detected and cultured are present in the petals of newly opened flowers [41]. Within the grasses, the ovaries are said to contain filamentous fungi, which can easily be isolated for further identification [42]. Such fungi can also be found in the pollen of forbs [43] early in the growth of flowers. Secondly, the number of microorganisms tends to increase as time progresses on individual blossoms. The most evident illustrations arise from the analysis of microorganisms that dwell on the pistil’s surface or are found in the nectar of the flower. An exponential increase was observed in a number of bacterial and fungal colonies in Datura wrightii once the blooming process started. Similar observations were recorded for *Agave palmeri*, which displayed minimal bacterial and fungal presence of microbial community in the nectar prior to blooming [40].

*Mimulus aurantiacus*, whose flowers are pollinated by hummingbirds, had a reported presence of yeast in one-fifth of flowers that were one day old. This number significantly increased as the flowers aged [44]. Likewise, there was a rise in both the frequency and quantity of bacteria found in the nectar of *Epilobium canum* [45]. The presence of animals visiting flowers leads to changes in the microbial communities found within the flowers. Despite the potential existence of microbes on flowers that show no signs of visitation, a significant amount of studies suggest that animal displays play a crucial part in transmitting the microbiota to and between floral parts. In 1884, Boutroux observed a higher prevalence of fungi (yeast) in flowers that were pollinated by bees [46]. Recent studies have shown that ascomycete flower yeasts are absent when bees and large-bodied pollinators are absent. Conversely, these yeasts are found in high quantities in the nectar of flowers imposed by pollinators [47]. Specific pollinator species can sometimes be implicated in flower dispersion [36]. In *Mimulus aurantiacus*, a plant pollinated by hummingbirds, flowers are more prone to the presence of bacteria in the nectar [45]. Nitidulid beetles vector particular species of large-spored ascomycete yeasts [48], and ants, florivores, and other non-mutualistic floral visitors have been shown to disperse flower-colonizing bacteria and yeasts [49].

**Figure 2:**
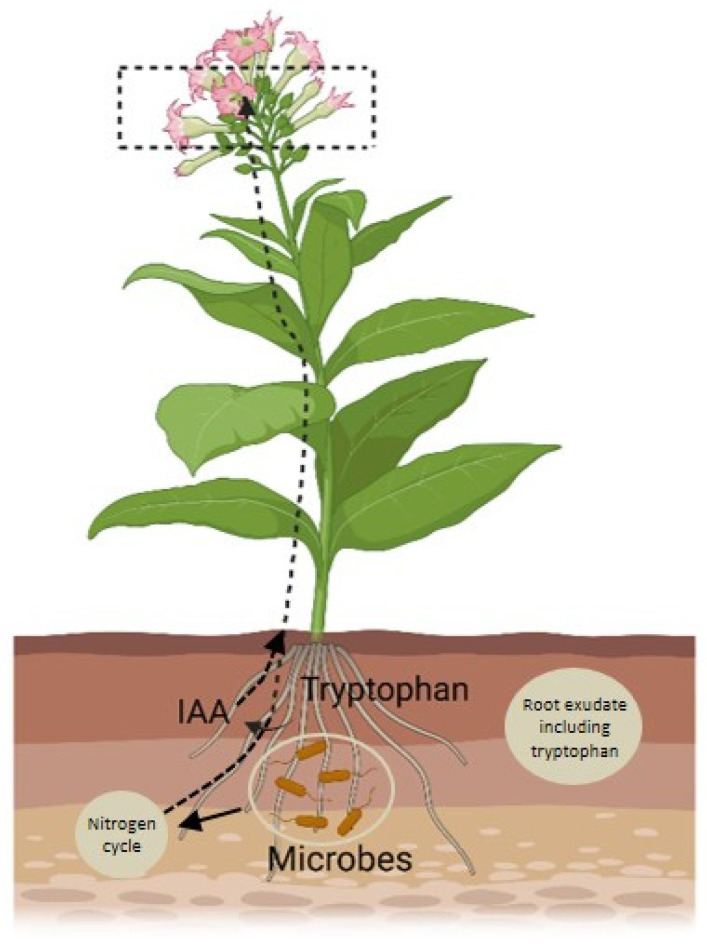
Schematic diagram illustrates the role of the rhizospheric microbiome in promoting floral development. The microbiome converts tryptophan into IAA (indole-3-acetic acid) and enhances the nitrogen cycle, both of which regulate floral development. The figure is adapted from the study reported by [53].

Apart from large insects and animals, small insects are also important vectors when it comes to the distribution of microbes residing in the nectar. For example, the microorganism that resides in flower-feeding thrips consists of *Rosenbergiella* and *Pantoea* [50]. More commonly, these microbes are present in parenthesis flowers [40] and pollen [51]. The evidence of dependency on the distribution of microbiota through animal vectors is more common in studies related to the microbiome of nectars. However, it does not necessarily mean that animal vectors do not contribute to microbial dispersion in other parts of the flowers. Bumble bees, for instance, are known to contribute to the dispersal of bacteria within stamens and petals of flower [52]. Other parts of flower-like corolla and leaves of the plants in close vicinity of the flowers have been reported for the presence of microbe-rich bee feces [38]. The presence of microbes in various floral parts suggests that microbes indeed play a significant role in flower development, either directly or indirectly. The underlying mechanisms, however, remain unexplored and require further investigation. Lu et al. (2018) recently investigated a network where root exudates influence rhizosphere microbes, which produce IAA (indole-3-acetic acid) and induce the nitrogen cycle, which further promotes flowering [53]. This creates a positive feedback loop where increased growth boosts exudation, further affecting flowering time (Figure 2).

## 2. Genetic Regulation of Flowering

### 2.1. Overview of Key Genes Involved in Flowering Time Regulation

For plants to successfully reproduce, blossoming at the right time is essential. It is controlled by a complex interaction between genetic pathways and environmental signals [54]. Several blooming processes are coordinated by genes such as Leafy (*LFY*), blooming Locus T (*FT*), and Suppressor of Overexpression of *CO1* (*SOC1*) [55]. *FT* genes play an important part in the regulation of time required for flowering in plants by interacting with various transcriptional regulators. These genes are members of the Phosphatidylethanolamine Binding Protein (PEBP) family and play a vital role in plant development [56]. Several species of plants that have *FT* genes are thoroughly investigated, suggesting their significance in stimulating inflorescence development and flowering [57]. The regulation of *FT* family genes in temperate grasses is influenced by the monocot-specific gene Indeterminate1 (*BdID1*), which affects the transition to flowering [58]. Different plant species have specific *SOC1* genes involved in flowering control. For instance, *Chrysanthemum morifolium cv*. *Jimba* expresses *CmSOC1*, which induces early flowering under short-day conditions by upregulating floral identity genes like *LFY* and *APETALA1* [59]. Three *SOC1* paralogs, *MtSOC1a*, *MtSOC1b*, and *MtSOC1c*, in *Medicago truncatula* provide overlapping roles in the transition from dormant to flowering. Triple mutants exhibited a phenotype of non-flowering [60]. *BrcSOC1*, found in pak choi, is overexpressed in *Arabidopsis thaliana* and causes a significant increase in flowering by positively impacting genes downstream, such as *AGAMOUS-LIKE 24* and *LFY* [61].

*LFY* genes are associated with flowering regulation in various plants, such as *DlLFY* in *Dimocarpus longan* [62] *PmLFY*-like in *Prunus mume* [63], and *MsLFY* in *Medicago sativa* [64] play crucial roles in regulating the timing of flowering in plants. These genes encode transcription factors that control floral growth and meristem differentiation. Overexpression of the *LFY* gene causes *Arabidopsis thaliana* to bloom and undergo morphological changes, suggesting that the gene functions similarly across all plant species. Through interactions with other proteins, including *ZFP4* and *TFL1*, *LFY* genes influence flowering processes. The floral repressors MADS Affecting Flowering 4 (*MAF4*) and *MAF5*, as well as the *LFY* gene, interact to regulate floral transition in *Arabidopsis thaliana*. *LDL1* and *LDL2* are essential in this interaction [65]. These interactions demonstrate the complex relationship of regulators that influence plant flowering processes.

### 2.2. Discussion of Major Flowering Pathways (Photoperiod, Vernalization, Autonomous Pathways)

**Figure 3:**
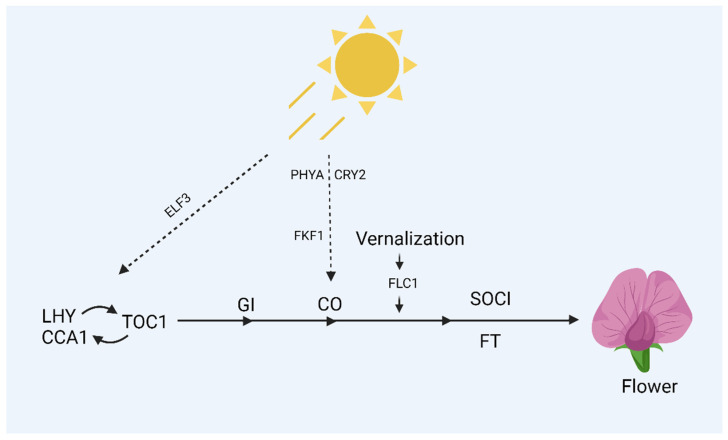
A molecule hierarchy regulating flowering in response to photoperiod. Arrows represent promotive effects between genes, while perpendicular lines represent repressive effects.

Both photoperiod and vernalization are known to affect flowering mechanisms in different plants. *Arabidopsis thaliana* flower in the spring or early summer when the days grow longer because of the plant’s resilient photoperiod sensitivity. Mutants with a reduced response to day duration were used to isolate mutations interacting with these responses [66]. These mutants were divided into two classes: those that flower under long days but not short ones and those that flower under short days but flower earlier than wild-type plants. Circadian rhythms are generally affected by some of the mutations that occur in early flowering during short days. In addition, about 6% of genes expressed properly are controlled by the circadian clock [67]. These circadian rhythms are generally affected by those types of mutations responsible for reducing response to day length. This, in turn, leads to early flowering in short days. These mutations generally impact genes like Early Flowering 3 (*ELF3*), Late Elongated Hypocotyl (*LHY*), Timing of Chlorophyll a/b Binding Protein 1 (*TOC1*), and the Circadian Clock Associated 1 (*CCA1*). Because the circadian clock is also involved in regulating the expression of these genes, mRNAs for *LHY* and *CCA1* only build up in the morning, and *ELF3* and *TOC1* only in the evening. The central mechanisms that produce circadian rhythms in plants may include *LHY*, *CCA1*, and *TOC1*. The expression pattern and sequence of *CCA1* and *LHY* are comparable, as described in Figure 3 [68], and have some genetic redundancy [69]. Circadian rhythms cycle more quickly, and plants blossom earlier in short days in the *LHY*, *CCA1* double mutant or the *TOC1* single mutant than in wild-type plants [70]. It was suggested that *LHY*, *CCA1*, and *TOC1* function together in a transcriptional feedback loop whereby the only expressed *TOC1* at night promotes the expression of *LHY*/*CCA1* at dawn, suppressing the expression of *TOC1* [69]. The circadian rhythms were reported to stop when *ELF3* mutants were exposed to prolonged photoperiods or continuous light [71], signifying the role of photoperiod in blossoming. Squamosa Promoter-Binding (*SPB*) genes, influenced by the circadian clock, play an important part in regulating the shift between vegetative growth and flowering. Additionally, miR156 and miR172 are involved in controlling some *SPB* genes during the flowering process [72].

Apart from photoperiod, vernalization also plays a critical role in the transition from the vegetative stage to flowering. Vernalization is the process of prolonged contact with the winter’s cold to promote the blossoming of plants [73]. The expression of a gene that codes for flowering repression is reduced in plants during vernalization. On the other hand, prolonged exposure to cold causes epigenetic modifications in the winter that last until the spring, which speeds up the next year’s flowering [73,74]. According to a study, the primary components involved in blooming time regulation by vernalization are *FLC* and FRIGIDA (*FRI*). Both *FLC* and *FRI* reduce flowers. By upregulating *FLC* expression, *FRI* negatively affects the length of blooming time [75]. The *FRI* gene is functional in winter-annual *Arabidopsis thaliana* plants. At the same time, it has been eliminated genomically in summer-annual plants [76], resulting in summer-annual plants blossoming earlier. Plants express high levels of *FLC* before vernalization to inhibit flowering. Winter cold exposure over an extended period suppresses the production of *FLC*, which releases *SOC1* and *FT* [77]. For the following spring’s floral transition, consistent suppression of FLC through vernalization is essential. *FLC* expressions are suppressed during vernalization due to epigenetic control. Polycomb Group Proteins (PcG) regulate epigenetic modifications that underline the repression of *FLC* through vernalization [78]. Histone H3 Lysine 27 Trimethylation (H3K27me3) develops during vernalization on enrichment of the Polycomb Repressive Complex 2 (PRC2) complex at FLC chromatin [79]. H3K27me3 increases at *FLC* because of VAL1 and VAL2′s direct interaction with the PRC2 complex to bring PHD-PRC2 to *FLC*. The *FLC* locus produces noncoding RNAs that support *FLC* silencing in addition to the PHD2-PRC2 complex [80].

### 2.3. Summary of Current Understanding of Genetic Regulation in Ornamental Plants

Recent investigations of genetic control in ornamentals focus on four areas: genomics, gene editing, molecular breeding, and polymerization for the improvement of some ornamentation traits (such as scent, color, flower shape, etc.) [81]. The molecular mechanisms of flowering in the ornamental species, where floral and fragrance phenotypes are under strict genetic control, are associated with flower development pathways, gene expression profiles, and the chemistry behind the scent aromas that can be modified utilizing biotechnological approaches [82]. Genetic engineering and genome-editing techniques have been widely used in the genetic manipulation of ornamental plants for trait improvement, including transgenic technology to produce blue flowers and CRISPR/Cas9-mediated precise gene editing [83]. In addition, special promoters are necessary for their expression in transgenic plants and overall improvements in the discovery of new types of cultivars [83]. Understanding disease resistance’s genetic and epigenetic regulation is important to help improve their genetic resources and develop resilient plant varieties through breeding and transgenics [84].

## 3. Hormonal Regulation of Flowering

### 3.1. Role of Key Hormones in Flowering

The flower indicates a change in the developmental stage of the plant from the vegetative to the reproductive stage [85]. Plant hormones, such as GA, ABA, cytokinin, ethylene, and auxin, have been identified as regulators of various developmental processes, including seed germination, plant growth, senescence, and flowering. The precise involvement of plant hormones in controlling the flowering process has long been a subject of debate [86] however, the literature suggests that endogenous hormones play an active role in flower bud differentiation [87] and the development of flower organs [88]. Auxin was previously noted as a crucial regulator of floral growth, but until recently, its function in flower development was unclear [89]. Auxins may impact floral initiation by inhibiting stem cells’ pluripotency or stimulating the genes that generate floral organ primordia [90]. Auxins significantly regulate flowers and floral organs [89]. Development of ovule and stamen in *Hyacinthus* from explants of perianth is influenced by different ratios of auxin and cytokinin [91]. ABA hormone in plants serves an important function in flowering. Several studies involving ABA mutants suggest a detrimental impact on Arabidopsis flowering [92]. Saffron has been reported to be negatively impacted by ABA in terms of induction of flowering [93].

Apart from auxin and ABA, GA has also been reported to play a critical role in flowering. It has been demonstrated that GA regulates all flowering stages, including floral growth and induction [94]. GA has been positively correlated with the induction of flowering in numerous species of plants, such as Arabidopsis, Nicotiana, and radish (*Raphanus sativus*) [86]. GA regulates the initiation and development of the flowers, which is critical for the fertility of males and females [95]. GA-deficient mutants in *Solanum lycopersicum* and *Arabidopsis thaliana* displayed abnormalities in development of stamen [96]. On the other hand, extreme GA deficiency resulted in sterility in female flowers [97]. The application of GAs and GA9 (a precursor of GA) helps in restoring the normal development of flowers. GA is also known for its role in corolla development in *Glechoma hederacea* [98]. Hu [99] identified stamens or flower receptacles as two potential sites for bioactive GA synthesis in *Arabidopsis thaliana* flowers and suggested that GAs are transported from these organs to promote petal growth. GA-deficit mutants produced short stamen and short filaments. The capability of self-pollination was also compromised in these plants [100]. In coordination with JA, GA also regulates late stamen development. In contrast, GA alone mediated early anther development [101].

In addition to model plants, the role of GA in the process of flowering has been investigated in various geophytes, including *Tulipa gesneriana*, *Anemone coronaria* L., *Zantedeschia aethiopica*, *Allium sativum*, Genus Lilium, Saffron, Paeonia and Hyacinthus, etc. [102]. Within the genus Zantedeschia, GA stimulates the initiation and development of floral growth, with the number of flower clusters contingent upon the specific dosages and duration of GA application [103]. Various studies on saffron have further identified that GA plays a contrary role in the control of flowering [93]. GA may decrease floral initiation, as reported in a study that investigated the signaling mechanism of hormones during the induction of flowering and its subsequent developmental stages [104]. GA is important in elongation of the pollen tube and germination of pollen [105]. The study also revealed non-germination of pollen in mutant varieties that could not produce GA. Upon supplementation with exogenous GA, the same plants resulted in germination of pollen [105]. A study revealed up to a seven-fold increase in GA content of the pollens during the growth of pollen tubes. However, this may only be limited to certain species. In plant species like Lilium and *Petunia hybrid*, the GA content was 200-fold more in the pollen when compared to GA content of the ovarian *tissue* [106]. Treatment of conscious and hermaphroditic lines in *Cucumis sativus* with exogenous GA3 supplementation promotes male tendency [107]. In an experiment on female *Cucumis sativus* lines, it was reported that consistent supplementation of GA3 could prevent the continuous female phase [108]. On the other hand, experiments on *Momordica charantia* L. revealed improved fruiting and female flower induction upon exposure to low concentrations of GA3 [109]. From the above discussion, it may be proposed that plant hormones play unique roles that vary from species to species at various phases of the development of flowers.

### 3.2. Interaction Between Hormones and Genetic Pathways in Flowering Regulation

A complex interplay between hormonal signals and genetic pathways governs the regulation of the blooming process, ensuring that flowers bloom at the optimal time to maximize reproductive success [110]. In *Arabidopsis thaliana*, auxins have been demonstrated to control the expression of the genes APETALA 1 (*AP1*) and *LFY* during floral initiation [90]. Auxin has been reported to promote flowering in strawberries (*Fragaria ananassa*). This is achieved through the expression of *FveARF4*, which is controlled by auxin. The expressed *FveARF4* binds to the regulators of the floral meristem recognition genes *FULL (FUL)* and *APETALA1 (AP1)* and triggers their expression, which in turn promotes blossoming in strawberries [111]. In addition to controlling plant flowering, *ARF* is also involved in controlling floral abscission. During the early stages of rose development, *RhARF7* regulates petal abscission [112]. The local distribution of auxins can be modulated by certain transcription factors. This is achieved through the regulation of gene expression in genes like *YUC* and *PIN* [113]. *YUCCA1* and *PIN1* genes could be detected in the developing flower head in Asteraceae flower head formation studies [24]. Auxin (*ARF3*) was discovered to regulate the *AGAMOUS* and *APETALA2* genes of flower organs passing through the developing stage [114]. Apart from auxins, cytokinins are considered to play an active part in the induction of flowering of several plant species. BAP has been reported to trigger the paralogs of *FT* genes to promote blooming in *Arabidopsis thaliana* [115]. In roses (*Rosa indica*), cytokinin stimulates flowering growth [116]. Cytokinin is also involved in strawberry flowering, where high levels of cytokinin are produced and transported to axillary buds. The auxin production thereby is inhibited in axillary buds due to high concentrations of cytokinins, which results in overcoming the dormancy [117,118]. Cytokinin also promotes flowering in saffron. This is achieved by cytokinin-modulated increases in *LFY* expression [119]. The function of *APETALA1* in developing floral meristems in *Arabidopsis thaliana* is further controlled by the cytokinin pathway [120]. In short-day conditions, the GA pathway supports the activation of the *SOC1* gene, which encourages flowering; however, this pathway does not affect the regulation of other flowering-related genes, such as *FLC* and *FT* [121]. At the same time, another study by [122] suggested reduced levels of GA2ox, an inhibitor of the GA pathway, and increased GA accumulations, indicating a beneficial role for GA in stimulating blooming. ABA regulates *FT* gene transcription through *GI*, *CO*, and *SOC1* expressions. However, its specific mechanism of action to regulate flowering is unclear [123].

### 3.3. Hormonal Crosstalk and Its Impact on Flowering Induction

Plant hormones, i.e., GA, ABA, auxins, cytokinin, and ethylene, have been abundantly reported in a number of studies to take part in the regulation of several developmental processes; however, a significant research gap exists when it comes to the mechanism of regulation of the process of flowering. In plants like *Raphanus sativus*, *Nicotiana*, and *Arabidopsis thaliana*, GA has been linked to the induction of flowering [124]. On the other hand, in citrus plants, *Vitis* spp., and apples (*Malus pumila*), GA has been reported to have an inhibitory effect on flower induction [125]. Besides the model plants, species like saffron, lilies, *Zantedeschia*, tulips, *garlic*, *Hyacinthus*, *lilium*, and *Anemone*, etc., have also been explored for the potential of GA in regulating flowering [102]. Promotion of initiation and development of flowers in *Zantedeschia* and the inflorescence number is regulated upon exposure to GA. However, the exact nature of the effect depends upon exposure time and dosage [103]. Varieties of *Arabidopsis thaliana* that need low temperatures for flower initiation have been reported to induce flowers even at high temperatures when supplemented with exogenous GA [126]. The contradictory impact of GA in terms of flower induction in saffron has also been indicated in the literature. Renau-Morata et al. (2021) [93] suggested inhibitory role of GA in flower initiation and development; however, in another study, evidence suggests that the downregulation of a GA-pathway inhibitor like GA2ox promotes flower induction [122]. It is therefore pertinent to further explore the exact role of GA in terms of regulation of processes involving initiation of flowering and its subsequent development.

Along with GA, another essential plant hormone, i.e., ABA, also impacts flowering in plants. In *Arabidopsis* species, ABA mutant varieties have displayed a negative impact in terms of flowering [92]; however, the evidence is not enough to elucidate a particular mode of action when it comes to the regulation of flowering upon exposure to ABA. Studies depict that ABA is responsible for regulating the transcription of *FT* gene by controlling the expression pattern of *SOC1*, *GI*, and *CO* [123]. Renau-Morata et al. (2021) [93] suggested the inhibitory role of ABA in flowering induction in saffron. Cytokinins on the other hand, have been reported to positively influence the induction of flowering in a number of plants, including *Arabidopsis thaliana* [120]. A similar positive role of cytokinin in flower induction was also reported in a study on rose plants [116] and strawberry [117]. Cytokinins are also known to regulate the functioning of *APETALA1* in order to establish floral meristems in *Arabidopsis* [120]. The auxin-to-cytokinin ratio plays a significant role in the ovule and stamen development from explants of parianth in *Hyacinthus* [91]. In a nutshell, it can be suggested that the role of plant hormones in various developmental stages of flowering is highly species-specific.

### 3.4. Recent Advances in Hormone Research and Their Implications for Ornamental Plant Breeding

Ornamental plants are an essential part of the horticultural industry, contributing significantly to the economy by producing and selling flowers, shrubs, and other decorative [127]. A primary area of interest for researchers has been breeding these plants to improve desired characteristics, including color, size, aroma, and stress tolerance [128]. Since hormones are essential for controlling a plant’s growth and development, new directions in hormone research have been explored in breeding ornamental plants [129]. Recent research has delved deeper into the hormonal pathways and their interactions within plants [130]. The development of synthetic hormones and growth regulators has provided new tools for breeders to influence plant growth and development [131]. Compounds that mimic natural hormones can induce rooting, flowering, and fruiting in ornamental plants, enhancing their esthetic appeal and market value [132]. By manipulating hormone pathways, breeders can enhance traits such as flower size, color, and longevity. For example, modifying ethylene sensitivity in ornamental flowers can delay senescence, resulting in longer-lasting blooms [133]. According to Chandler [134], plants can become more visually appealing by enhancing flower symmetry and branching patterns through hormonal regulation. Hormones are crucial in plant responses to abiotic stresses such as drought, salinity, and temperature. Advances in hormone research have led to ornamental plants with enhanced stress tolerance [135]. For instance, overexpressing genes involved in ABA biosynthesis can improve drought tolerance, making ornamental plants more resilient in various climates [136].

Hormones are integral to in vitro propagation techniques and are widely used in the ornamental plant industry for mass production [137]. Understanding the hormonal requirements for tissue culture has enabled the efficient propagation of numerous ornamental species, ensuring uniformity and quality in the final product [137]. Hormonal research has facilitated overcoming hybridization barriers in ornamental plant breeding [138]. For example, cytokinins can promote cell division and development in hybrid embryos, increasing the success rate of interspecific crosses [139]. Advanced breeding programs now incorporate hormone treatments to synchronize flowering times, enhancing the efficiency of hybridization efforts. Studies on ethylene, a hormone associated with fruit ripening and flower senescence, have led to the development of ethylene-resistant varieties. These varieties exhibit prolonged flower life, a trait that is highly desirable for cut and potted plants [140]. Research on GA has enabled the creation of dwarf varieties of ornamental plants [141]. These compact plants are popular in urban gardening and landscape design due to their manageable size and reduced maintenance needs [142]. Advances in auxin research have improved the rooting efficiency of cuttings, a common propagation method in the ornamental plant industry. Enhanced rooting leads to higher propagation success rates and better plant establishment [143].

## 4. The Microbiome and Flowering Induction

### 4.1. Introduction to the Plant Microbiome and Its Importance in Plant Health and Development

The interaction between plants and the wide variety of microbes contributing to their microbiome is essential to their growth and development, as shown in Table 1. Microorganisms play significant functions in these symbiotic relationships, assisting plants by increasing nutrient intake, stimulating hormones, and improving stress tolerance [144]. While it impacts plant health through interactions between microbes and plants, the rhizosphere, the area around plant roots, is particularly important for these interactions [145]. Additionally, plant microbiomes may contain plant growth-promoting bacteria (PGPB), which promote plant development and function as biocontrol agents to neutralize phytopathogens, improving crop fitness in agriculture [146]. For example, endophytes colonize various crop parts and benefit the hosts, rhizobia fix nitrogen for legumes, and mycorrhizae assist in nutrient absorption [147]. Sustainable agriculture depends upon comprehending and utilizing these complex plant–microbe interactions, illustrating the need for further research and useful applications in agricultural biotechnology [144].

### 4.2. Evidence Suggesting a Role of the Microbiome in Flowering Induction

Studies involving the exploration of plant–microbe interaction have demonstrated the critical function of the microbiome in flowering induction. According to studies, when flowers are exposed to phytopathogens like *Erwinia amylovora*, their microbiome, especially the stigma, exhibits dynamic changes that influence the development of disease [163]. Furthermore, it has been found that nectar-dwelling bacteria, including Acinetobacter, take advantage of pollen nutrition by stimulating pollen germination and bursting, suggesting a direct microbial influence on pollen physiology and possibly on plant reproduction [164]. In addition, recent research discovered that insect-vectored bacteria can genetically modify plant cells to produce complex structures like galls. This demonstrates how microorganisms can control plant development to produce unique interactions between insects and plants [165]. Studies have demonstrated that the microbial communities in the rhizosphere can control the time of flowering by influencing the availability of nitrogen and the generation of phytohormones [53]. Moreover, plant-associated microbial communities, particularly floral microorganisms, can change the aroma of plants and flowers, thus changing insect reactions and possibly pollination [166].

### 4.3. Mechanisms by Which Microbiota May Influence Flowering Time (e.g., Hormone Production, Nutrient Availability)

Microbiota can influence flowering time through multiple mechanisms. Research has demonstrated that rhizosphere microbial communities can regulate the timing of flowering by modulating nutrient availability and hormone signaling pathways, which affect the gene expression associated with flowering time [53]. For example, studies on plants like *Arabis alpina* have shown that root microbiota plays a crucial role in determining flowering time, with factors such as soil type and the duration of plant residence in the soil being significant determinants of root microbiota variation [167]. Additionally, soil microbial communities have been identified as key influencers of flowering time, with different soil microbiota altering selection patterns and contributing to the phenotypic plasticity of flowering time in plants like *Boechera stricta* [168]. These interactions between microbes and plants impact various phenological transitions, highlighting the significant role of microbiota in nutrient acquisition, phytohormone signaling, and gene expression related to flowering time.

## 5. Interconnection Between Endogenous and External Factors

### 5.1. Integration of Findings from Endogenous and External Factors Studies to Provide a Holistic Understanding of Flowering Induction

This review paper thoroughly explains flowering induction by investigating microbiome, hormones, and genetics studies. Genetically, blooming is regulated by a network of genes that react to various internal and external stimuli. Important genes such as *SOC1*, blooming LOCUS T (*FT*), and *CO* flowers are primarily responsible for the photoperiodic regulation of blooming [169]. Meanwhile, CO, a crucial regulator, promotes *FT* expression in response to long-day conditions. Ultimately, the FT protein proceeds from the leaves to the shoot’s apical meristem, interacting with *SOC1* to initiate the flowering process [170]. *FLC*, a floral regulator gene, is epigenetically inhibited by *FT* and *SOC1* triggering, resulting in vernalization, which promotes flowering [171]. Plants perform a complex process of induction of blooming, which is regulated by hormone signals, genetic pathways, and environmental factors. The photoperiodic, vernalization, autonomous, and GA pathways are among the important genetic pathways connected to this process that have been identified through research on *Arabidopsis thaliana* and other plant species [172]. Furthermore, hormone regulation significantly influences blooming control, even though this pathway has not historically been considered important. Hormonal pathways may potentially mediate stress signals [173]. The formation of flower buds in pears has been associated with regulating the circadian clock by specific genes, such as *ELF3*, demonstrating the genetic foundation of blooming [174]. Specific microbial populations can produce plant hormones or hormone-like substances that affect flowering. For instance, rhizobacteria that generate the IAA might impact blooming indirectly by influencing the hormonal balance of the plant to promote root growth [175]. Integration of information obtained from genes, hormones, and microbiome is essential for controlling blooming. Hormonal signals can influence the genetic pathways that regulate flowering, and the microbiome can potentially impact these processes [176].

### 5.2. Implications for Ornamental Plant Breeding and Cultivation Practices

In recent years, there has been a major change in the breeding and cultivation of ornamental plants, with an increase in the application of biotechnological technologies such as genome editing and genetic engineering to enhance particular traits like unique flowers [177]. By methods like somatic embryogenesis and organogenesis, in vitro cultures are essential for propagating ornamentals, providing solutions to the lack of plant materials, and boosting diversity [137]. Sustainability is a significant problem in the production of ornamentals. It aims to lessen adverse environmental effects by altering cultivation practices such as integrated fertilizer management and the use of recyclable materials [178]. Genetic engineering and interspecific hybridization are techniques used to address abiotic stress tolerance in ornamental crops, leading to the development of high-quality, stress-tolerant ornamental varieties [179]. For breeders seeking to commercialize genetically modified or gene-edited ornamental plants, challenges exist despite the developments, including the need for international harmonization in genetic modification regulations and regulatory challenges [180].

### 5.3. Future Directions for Research in Understanding the Complex Networks Underlying Flowering in Ornamental Plants

Analyzing gene co-expression networks to identify the genetic mechanisms influencing floral variety may be the next step in investigating the intricate networks behind the flowering of ornamental plants [181]. Examining the regulatory networks associated with flower development, particularly in species like *Chrysanthemum morifolium*, can provide insight into the mechanisms controlling the determination of floral morphology [182]. More understanding of the many gene interactions influencing important agronomic characteristics might be realized with further investigations into the molecular basis of photoperiodic flowering regulation, as demonstrated in soybeans, using sophisticated network inference techniques like CausNet [183]. Combining transcriptome data with network analysis techniques might enable further studies to identify the important genes, regulatory relationships, and modules that control the blossoming processes of ornamental plants. This allows for breeding and genetic modification strategies to be more specific.

## 6. Summary and Future Prospects

**Figure 4:**
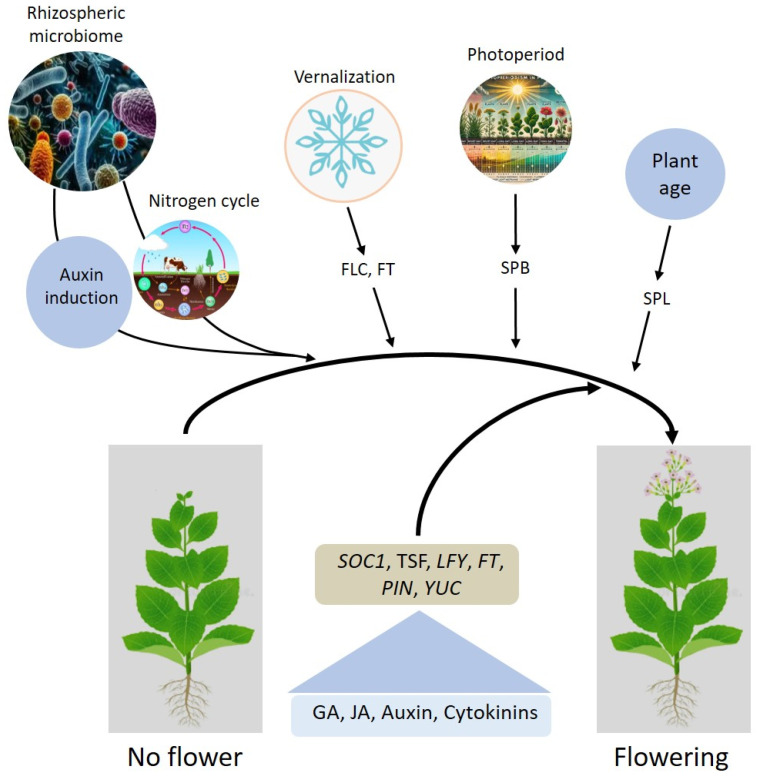
Factors regulating the transition from vegetative to flowering stages in plants. Various environmental and developmental signals, including the rhizospheric microbiome, nitrogen cycle, auxin induction, vernalization, photoperiod, and plant age, converge to regulate flowering. Key pathways and genes involved include *FLC* (FLOWERING LOCUS C) and *FT* (FLOWERING LOCUS T) for vernalization, *SPB* (SUPPRESSOR OF PHYTOCHROME B) for photoperiod, and *SPL* (SQUAMOSA PROMOTER BINDING PROTEIN-LIKE) for plant age. These signals modulate the expression of central flowering regulators such as *SOC1* (SUPPRESSOR OF OVEREXPRESSION OF CONSTANS1), *TSF* (TWIN SISTER OF FT), *LFY* (LEAFY), *FT*, *PIN* (PIN-FORMED), and *YUC* (YUCCA). Hormones like gibberellic acid (GA), jasmonic acid (JA), auxin, and cytokinins further facilitate the transition from vegetative growth to flowering.

The regulation of flowering in plants is a multifaceted process governed by intricate interactions between endogenous and external factors, as well as environmental cues such as vernalization and photoperiodism. Genetic pathways, including photoperiod and vernalization pathways, play a pivotal role in transitioning plants from vegetative to reproductive stages. Key genes, such as *FT*, *FLC*, *LFY*, and *SOC1*, coordinate responses to these external signals, with vernalization epigenetically repressing flowering inhibitors like *FLC* to facilitate bloom induction in response to prolonged cold exposure. Similarly, photoperiodism, regulated by circadian-controlled genes like *SPB* and downstream effectors, aligns flowering with seasonal and day-length changes to maximize reproductive success. Hormones such as GA, auxins, and cytokinins modulate flowering by influencing meristem size, organ differentiation, and developmental timing. Hormonal crosstalk is crucial in integrating genetic signals with environmental stimuli, while the microbiome plays an emerging role by producing hormones like IAA and enhancing nutrient availability, further influencing flowering time and quality. Despite recent advancements, gaps remain in our understanding of how these systems interact holistically. For instance, the precise molecular and epigenetic mechanisms integrating photoperiod and vernalization pathways with hormonal and microbiome inputs are not fully elucidated. The summary of this study is illustrated in Figure 4. Future research should use high-resolution multi-omics approaches to unravel the integration of vernalization and photoperiod pathways with endogenous (genetic) and external reasons (biotic and abiotic stress) interactions. Investigating microbiome contributions to flowering regulation under diverse conditions can promote sustainable practices. Advanced biotechnological tools like CRISPR-Cas9 can tailor flowering traits and overcome seasonal limitations.

## Figures and Tables

**Table 1:** Beneficial microbes’ role in flowering growth.

Microbe	Type	Plant Host	Effect on Flowering	References
*Burkholderia phytofirmans*	Bacteria	*Arabidopsis thaliana*	Promotes flowering time	[148]
*Bacillus subtilis*	Bacteria	Marigold (*Tagetes*)	Flowering color	[149]
*Rhizobium* spp.	Fungi	*Mangifera indica* L. cv	Flowering number	[150]
*Fusarium oxysporum*	Fungi	*Arabidopsis thaliana*	Flowering timing	[151]
*Piriformospora indica*	Fungi	*Coleus forskohlii*	Promotes early flowering	[152]
*Trichoderma harzianum*	Fungi	Chrysanthemum	Flowering number	[153]
*Arbuscular mycorrhizal fungi*	Fungi	*Antirrhinum majus* L.	Promote flowering	[154]
*Azospirillum brasilense*	Bacteria	Petunia	Promotes flowering number	[155]
*Pseudomonas putida*	Bacteria	*Euphorbia pulcherrima*	Promotes flowering number	[156]
*Glomus intraradices*	Mycorrhizal fungi	*Crossandra infundibuliformis* L.	Promote flowering weight and number	[157]
*Bacillus* spp.	Bacteria	*Solanum lycopersicum*	Flowering time	[158]
*B. acidiceler*, *B. subtilis* and *B. pumilus*	Bacteria	*Rosa hybrida* L.	Flowering diameter	[159]
*Pseudomonas* spp.	Bacteria	*Petunia* plants	Flower number	[160]
*Bacillus subtilis* FZB24	Bacteria	*Crocus sativus*	Flowers per corm	[161]
*Trichoderma inocula*	Fungi	*Petunia* plant	Promotes flowering	[162]

## Data Availability

The data are available in the manuscript.
